# Soluble PD-L1 improved direct ARDS by reducing monocyte-derived macrophages

**DOI:** 10.1038/s41419-020-03139-9

**Published:** 2020-10-30

**Authors:** Jing Xu, Jiahui Wang, Xiaoli Wang, Ruoming Tan, Xiaoling Qi, Zhaojun Liu, Hongping Qu, Tingting Pan, Qingyuan Zhan, Yong Zuo, Wen Yang, Jialin Liu

**Affiliations:** 1 Department of Critical Care Medicine, Ruijin Hospital, Shanghai Jiao Tong University School of Medicine, Shanghai, China; 2 Department of Biochemistry and Molecular Cell Biology, Shanghai Jiao Tong University School of Medicine, Shanghai, China; 3 Department of Pulmonary and Critical Care Medicine, China-Japan Friendship Hospital, Beijing, China

**Keywords:** Respiration, Prognostic markers

## Abstract

Acute respiratory distress syndrome (ARDS) is common in intensive care units (ICUs), although it is associated with high mortality, no effective pharmacological treatments are currently available. Despite being poorly understood, the role of programmed cell death protein 1 (PD-1) and PD-ligand 1 (PD-L1) axis in ARDS may provide significant insights into the immunosuppressive mechanisms that occur after ARDS. In the present study, we observed that the level of soluble PD-L1 (sPD-L1), a potential activator of the PD-1 pathway, was upregulated in survivors of direct ARDS than in non-survivors. Administration of sPD-L1 in mice with direct ARDS relieved inflammatory lung injury and improved the survival rate, indicating the protective role of sPD-L1 in direct ARDS. Using high-throughput mass cytometry, we found a marked decrease in the number of lung monocyte-derived macrophages (MDMs) with proinflammatory markers, and the protective role of sPD-L1 diminished in ARDS mice with monocyte/macrophage depletion. Furthermore, PD-1 expression increased in the MDMs of patients and mice with direct ARDS. Finally, we showed that sPD-L1 induced MDM apoptosis in patients with direct ARDS. Taken together, our results demonstrated that the engagement of sPD-L1 on PD-1 expressing macrophages resulted in a decrease in pro-inflammatory macrophages and eventually improved direct ARDS. Our study identified a prognostic indicator for patients with direct ARDS and a potential target for therapeutic development in direct ARDS.

## Introduction

Acute respiratory distress syndrome (ARDS) is a common disease in intensive care units (ICUs) caused by various pulmonary or extrapulmonary factors. The morbidity of ARDS accounts for 10% of ICU admissions and has a high mortality (34.9–46.1%)^1^. Despite this high mortality rate, the pathogenesis of ARDS is not well-understood and there were no effective pharmacological treatments. The immune responses play a pivotal role in the process, in which recruited pro-inflammatory immune cells are the main contributors to the pathophysiology and mechanisms of injury that lead to ARDS^2^.

Programmed death-1 (PD-1) and its ligand PD-L1, the most studied immunosuppressive proteins, were well-known for their roles in regulating adaptive immune cell components in various diseases^3^. Recently, PD-1/PD-L1 axis was demonstrated to mediate the functions of macrophages, thereby participating in some acute inflammatory diseases^4–6^. Genome-wide expression measurements revealed that the expression of PD-L1 and PD-L1/PD-1 pathway-associated gene were significantly upregulated in patients with ARDS who survived or were extubated within 28 days compared to non-survivors or intubated patients^7^. In contrast, blockade of the PD-1 pathway induced ARDS-like pneumonitis in patients during anti–PD-1/PD-L1 therapy^8,9^. More recently, Monagham et al. reported soluble PD-1 as a potential biomarker in human and experimental extrapulmonary ARDS^10^. These results suggest that the PD-1/PD-L1 axis is involved in the development of ARDS.

The soluble form of PD-L1 (sPD-L1) was unveiled to bind with PD-1 and the interaction reduced the CD3-TCR activation, therefore, was considered as the potential activator of PD-1 pathway^11,12^. It was identified to be a novel molecule that retained the ability of immune regulation independently of cell-cell contacts^11^^[,13^^[,14^. Here we reported for the first time that sPD-L1 was associated with the mortality of patients with direct ARDS and played a protective role in the corresponding experimental models. To identify the target cells of sPD-L1 in direct ARDS, we used high-throughput mass cytometry (cytometry TOF, CyTOF) for high-dimensional analysis of cell surface markers, signaling molecules and cytokines in mouse lung immune cells at the single-cell level^15,11^. A specific cluster of MDMs with high expression of CCR2^+^ and M1-like macrophage markers was detected to be markedly reduced after administration of sPD-L1, and the protective role of sPD-L1 in direct ARDS diminished in mice with monocyte/macrophage depletion. Finally, mechanistic studies showed that sPD-L1 induced the apoptosis of monocyte-derived macrophages (MDMs) in patients with direct ARDS.

Overall, our study suggested that sPD-L1 played a protective role in direct ARDS by reducing the quantity of a specific cluster of lung MDM. The mechanism underlying this effect was related to MDM apoptosis. These findings provided an insight into immune regulation in direct ARDS via the PD-1/PD-L1 pathway and suggested a new potential drug target for the treatment of ARDS.

## Results

### Low levels of serum sPD-L1 indicated more severe disease and predicted worse prognosis in patients with direct ARDS

A total of 43 patients who fulfilled the Berlin definition of ARDS were included in this study between May 2018 and November 2019. Mechanical ventilation was reported to be associated with the expression of PD-L1^7^. Therefore, 20 non-ARDS patients receiving mechanical ventilation and 10 healthy volunteers were included as controls. The demographic and clinical data of the patients included in this study are shown in Table S1. No significant differences were found in age or sex among the ARDS patients, non-ARDS patients, and healthy controls.

**Fig. 1:**
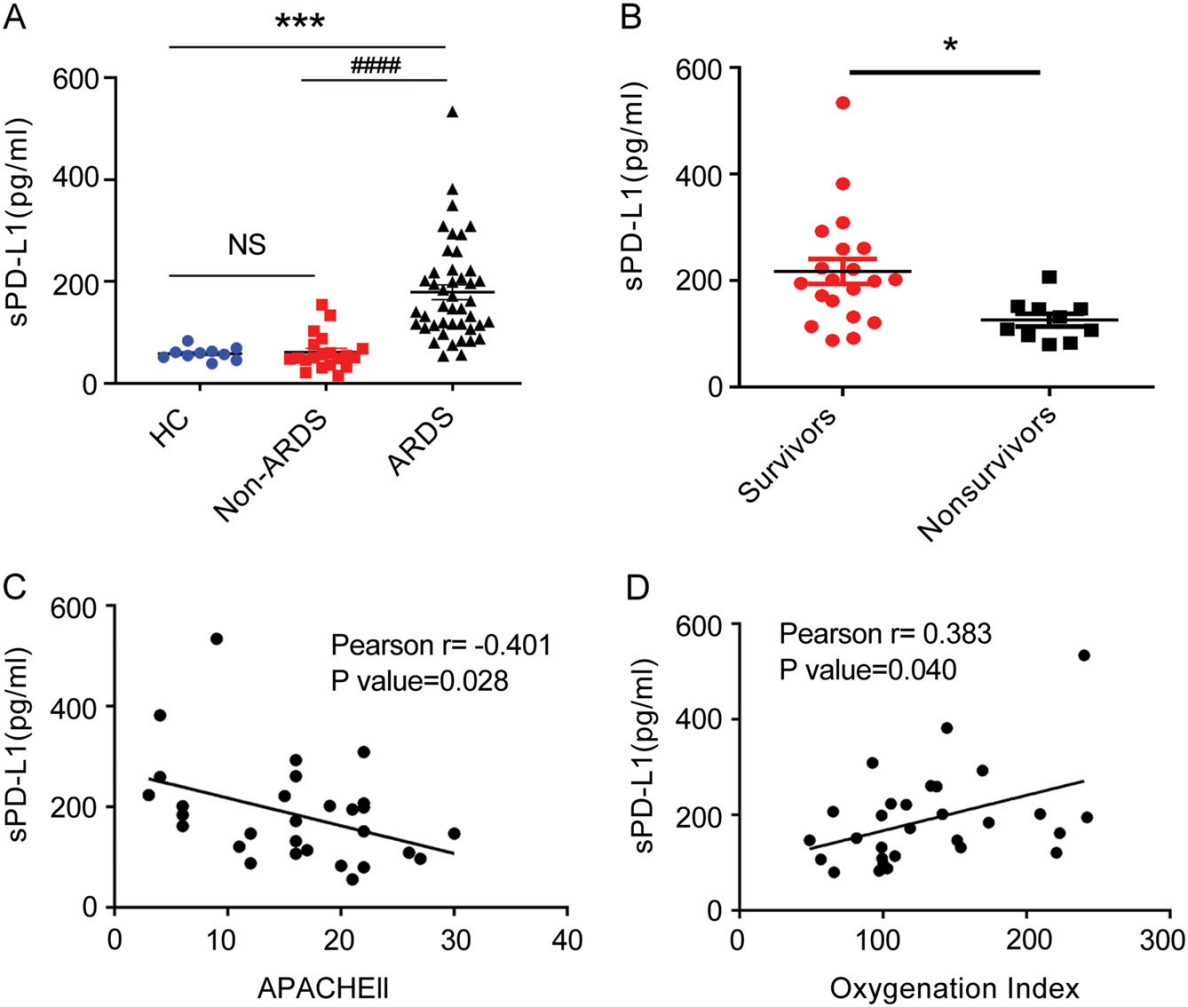
Low levels of serum sPD-L1 indicated more severe disease and predicted worse prognosis in patients with direct ARDS. **A** Serum sPD-L1 levels in healthy controls (*n* = 10), Non-ARDS patients with mechanical ventilation (*n* = 20) and ARDS patients (*n* = 44). **B** sPD-L1 levels of survivors (*n* = 20) and non-survivors (*n* = 10) of direct ARDS. **C** Correlations between APACHEII score and serum sPD-L1 levels in patients with direct ARDS (*n* = 30). **D** Correlations between oxygenation index and the levels of serum sPD-L1 in patients with direct ARDS (*n* = 30). *P < 0.05, **P < 0.01, ***P < 0.001, *P* value was determined by unpaired *t* test. ^####^*P* < 0.01, *P* value was determined by Man–Whitney test.

Serum sPD-L1 levels were significantly higher in ARDS patients than in the healthy controls and non-ARDS patients (Fig. 1A). The ARDS patients were categorized as survivors and non-survivors based upon ICU mortality (Table S1). sPD-L1 and soluble PD-1 (sPD-1) levels did not significantly differ between the survivors and non-survivors of ARDS (Fig. S1A, B). ARDS can be subclassified into direct and indirect types. Giving that the majority of our patients had direct ARDS, those with indirect ARDS were excluded to reduce heterogeneity. The demographic and clinical data of the patients with direct ARDS included in this study are shown in Table 1. Non-survivors of direct ARDS had higher acute physiology and chronic health evaluation (APACHE) II scores, higher leukocytes levels, lower oxygenation index (PaO_2_/FiO_2_) than the survivors. Other indicators, including age and sex were comparable between the two groups. Serum sPD-L1 levels were significantly lower in non-survivors than in survivors with direct ARDS (Fig. 1B) whereas sPD-1 levels had no significant change (Fig. S1A). We also detected the levels of other ARDS-related cytokines^12,13^. The levels of sPD-1, IL-10 and IL-17 had no significant difference between the survivors and non-survivors of direct ARDS (Fig. S1C–E). Receiver operating characteristic (ROC) curve analyses were used to assess the ability of serum sPD-L1 in predicting ICU mortality in patients with direct ARDS. The results highlighted a high prognostic accuracy (area under the curve [AUC] 0.808) of sPD-L1, which was comparable to the APACHE II score (Fig. S2).

**Table 1:** Demographics of direct ARDS patients and controls.

	Controls		Direct ARDS			
	Healthy controls	Non-ARDS	Total	Survivors	Non-survivors	*P* value
N	10	20	30	20	10	
Age, year	61.7 ± 11.5	63.4 ± 8.5	62.3 ± 12.6	61.3 ± 11.0	64.4 ± 15.9	NS
Gender, male, *n* (%)	7 (70.0)	15 (75.0)	23 (76.7)	15 (75.0)	8 (80,0)	NS
**Comorbidities,** ***n*** **(%)**						
*Cardiovascular disease*		8 (40.0)	10 (33.3)	5 (25.0)	5 (50.0)	NS
*Diabetes*		2 (10.0)	3 (10.0)	2 (10.0)	1 (10.0)	NS
*Malignancy*		3 (15.0)	1 (3.3)	0	1 (10.0)	NS
*Chronic respiratory disease*		1 (5.0)	4 (13.3)	1 (5.0)	3 (30.0)	NS
*Neurologic disease*		0	1 (3.3)	0	1 (10.0)	NS
*Gastrointestinal diseases*		8 (40.0)	4 (13.3)	3 (15.0)	1 (10.0)	NS
*Cerebral infarction*		0	4 (13.3)	0	4 (40.0)	<0.01
*Chronic kidney disease*		2 (10.0)	6 (20.0)	4 (20.0)	2 (20.0)	NS
*Kidney transplant*		0	2 (6.7)	2 (10.0)	0	NS
*Liver disease*		1(5.0)	4 (33.3)	4 (20.0)	0	NS
**Pathogens,** ***n*** **(%)**						
*Acinetobacter baumannii*				4 (20.0%)	1 (10.0)	NS
*Pseudomonas aeruginosa*				2 (10.0%)	1 (10.0)	NS
*Klebsiella pneumoniae*				3 (15.0)	3 (30.0)	NS
*Candida albicans*				1 (5.0)	0	NS
*Virus*				3 (15.0)	2 (20.0)	NS
*Undiagnosed pathogens*				7 (35.0)	3 (30.0)	NS
APACHE II		7.5 ± 3.3	15.7 ± 7.5	12.9 ± 6.8	21.3 ± 5.5	<0.01
PaO_2_/FiO_2_		273.5 ± 84.1	126.1 ± 50.4	143.2 ± 48	91.9 ± 36.8	<0.01
CRP, mg/dL		31.1 ± 44.4	84.5 ± 93.7	66.7 ± 67.5	120.2 ± 128.8	NS
PCT, ng/mL		0.66 ± 1.3	4.1 ± 8.5	4.5 ± 9.8	3.2 ± 5.4	NS
Leukocytes, ×10^9^/L		11.2 ± 4.3	12.3 ± 5.9	10.6 ± 4.7	15.5 ± 6.9	0.03
Neutrophils,×10^9^/L		9.3 ± 4.5	4.5 ± 4.7	3.7 ± 3.3	5.8 ± 6.4	NS
Lymphocytes,×10^9^/L		0.9 ± 0.8	0.4 ± 0.4	0.4 ± 0.4	0.4 ± 0.4	NS
VFD, days			15 ± 10.5	19.3 ± 8.7	6.6 ± 8.7	<0.01
ICU stay, days			42.6 ± 51.4	34.6 ± 41.3	57.8 ± 66.5	NS

Quantitative data are presented as mean ± SD, Qualitative data are presented as number (%), *P*-value for the survivors and non-survivors of direct ARDS.

*APACHE* acute physiologic and chronic health evaluation, *CRP* C-reactive protein, *PCT* procalcitonin, *VFD* Ventilator-free days.

A significant negative correlation was observed between the APACHE II score and the sPD-L1 levels in patients with direct ARDS (Fig. 1C). Serum sPD-L1 levels were positively correlated with PaO_2_/FiO_2_ (Fig. 1D).

### Administration of sPD-L1 attenuated inflammatory lung injury and improved survival rate in mice with direct ARDS

**Fig. 2:**
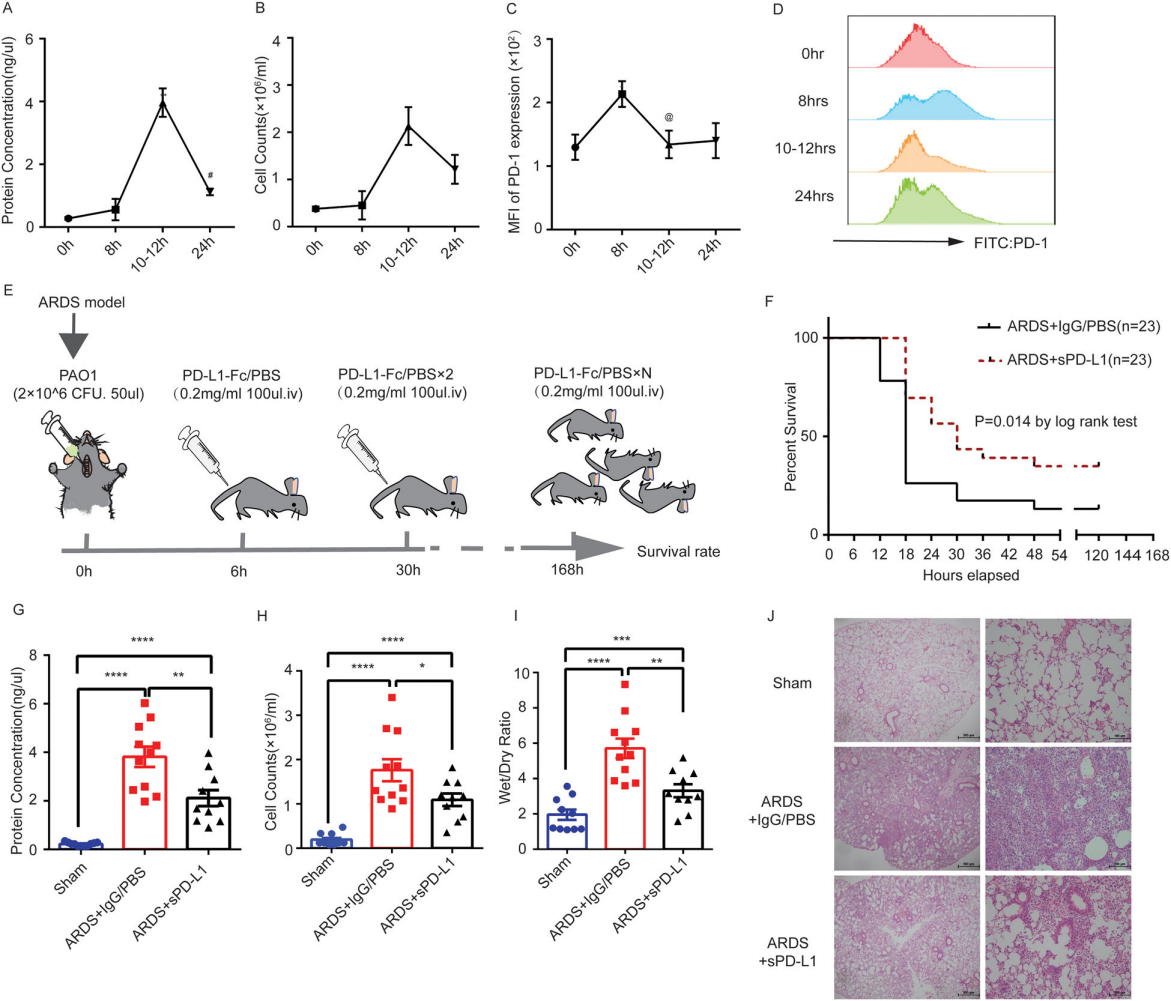
Administration of sPD-L1 attenuated inflammatory lung injury and improved survival rate in mice with direct ARDS. **A** Time course of protein concentration in bronchoalveolar lavage fluid (BALF) of ARDS mice (*n* = 5/each group). **B** Time course of cell counts in BALF of ARDS mice (*n* = 5/each group). **C** Time course of PD-1 expression in CD45 + lung cells of ARDS mice (*n* = 5/each group). **D** PD-1 expression in CD45 + lung cells of ARDS mice at different time points post-infection (*n* = 5/each group). **E** Schematic depicting experiment workflow: The mice were intratracheal injected with PAO1(2 × 10^6^ CFU/mL, 50 µL/mouse) to establish the ARDS model, 6 h later the mice were randomly divided into two groups. The mice in sPD-L1 group were intravenous injected with 20 µg/100 µl sPD-L1 protein while those in PBS group were administrated with 100 μL PBS. The mortality was monitored for 7 days and. Every 24 h the mice were administrated with another dose of sPD-L1 or PBS. **F** Kaplan–Meier plot of survival rate of ARDS mice (*n* = 23/group) *P < 0.05 by log-rank test. **G** To detect the lung injury after administration of sPD-L1/PBS, mice were sacrificed at 12-h post infection (6-h post administration of sPD-L1/PBS). Protein concentration in BALF of Sham mice, IgG/PBS-treated ARDS mice, and sPD-L1-treated ARDS mice (*n* = 10–11/each group). **H** Cell counts of BALF of Sham mice, IgG/PBS-treated ARDS mice, and sPD-L1-treated ARDS mice (*n* = 10–11/each group). **I** Wet to dry ratio of the left lung in Sham mice, IgG/PBS-treated ARDS mice, and sPD-L1-treated ARDS mice (*n* = 10–11/each group). **J** HE staining of the left lung in control, IgG/PBS-treated ARDS mice, and sPD-L1-treated ARDS mice (*n* = 10–11/each group) The experiments have been repeated for at least three times. Each value represents the mean ± SEM of three independent experiments. *P < 0.05, **P < 0.01, ***P < 0.001, analyzed by *t* test.

Clinical data provided insight into the protective role of sPD-L1 played in direct ARDS. Given that sPD-L1 was reported to retain its receptor-binding domain with PD-1 and could deliver immunosuppressive signals as membrane PD-L1, we used Fc-conjugated programmed death ligand 1 (PD-L1-Fc) protein to mimic sPD-L1 and investigate the function of PD-L1 in ARDS mice. The model of ARDS was prepared using wild-type C57BL/6 mice by intratracheal injection of PAO1 (Pseudomonas aeruginosa), and the time course of lung injury and PD-1 expression in the lung cells were observed to determine the time point of sPD-L1 treatment and tissue harvest. The severity of lung injury, evaluated by protein and white cell counts of BALF, increased during the first 8-h post-infection and peaked at 10–12 h (Fig. 2A, B), with PD-1 expression reaching a peak at 8 h (Fig. 2C, D). Therefore, based on experimental kinetics, mice were first treated with PAO1 (2 × 10^6^ CFU/mL, 50 µL/mouse) at 0 h, followed by delayed sPD-L1 treatment at 6 h to allow sPD-L1 distribution in the blood thoroughly for up to 8 h and binding to as many PD-1-binding sites as possible (Fig. 2E). To determine the distribution of sPD-L1 in lung, the His fused sPD-L1 protein was used as a tracer by intravenously injected into the ARDS mice 6-h post-infection. The sPD-L1-his protein evaluated by immunoblotting appeared in lung protein lysis at different time point (Fig. S4A), and the sPD-L1-his protein appeared on the surface of the lung cells (black arrows pointed to the sPD-L1-His protein) (Fig. S4B) revealed by immunohistochemistry, indicating that the injected sPD-L1 protein could locate to the lung. To verify the role of sPD-L1 in the mortality with ARDS, the mice in the sPD-L1 group were monitored for 7 d (Fig. 2E). In the phosphate-buffered saline (PBS) group, ARDS-associated mortality was ~87.0% (20 of 23 mice) at 7 day (Fig. 2F). The mice administered with sPD-L1 were significantly protected against ARDS induced lethality, only 65.2% of the mice died (15 of 23 mice) (Fig. 2F). Thus, sPD-L1 can efficiently protect mice from early death due to ARDS. Next, we investigated the mechanism underlying the contribution of sPD-L1 in reducing the mortality of patients with direct ARDS. The mice were sacrificed at 12 h to harvest the lung and BALF. sPD-L1 treatment significantly reduced the protein levels of BALF (a marker of permeability injury), white cell counts in BALF (the recruitment of immune cells) (Fig. 2G, H) and wet/dry ratio (lung edema) (Fig. 2I). In parallel to other indicators, lung morphology assessed by HE staining showed reduced lung edema, alveolar wall thickness, and the recruitment of pro-inflammatory cells in sPD-L1 treatment group (Fig. 2J). To further demonstrated sPD-L1 alleviated lung injury by reducing the lung inflammation rather than the bacterial load, we assayed the load of pseudomonas aeruginosa in BALF of ARDS mice treated with sPD-L1 or PBS. We found the bacterial load in two groups had no significant differences (Fig. S5A). Moreover, the levels of TNF-α in BALF were determined by ELISA kit, the administration of sPD-L1 significantly reduced the levels of TNF-α of ARDS mice (Fig. S5B). These experiments suggested that sPD-L1 reduced inflammatory lung injury of mice with direct ARDS, which may contribute to the reduced mortality.

### CyTOF revealed that administration of sPD-L1 reduced MDMs

**Fig. 3:**
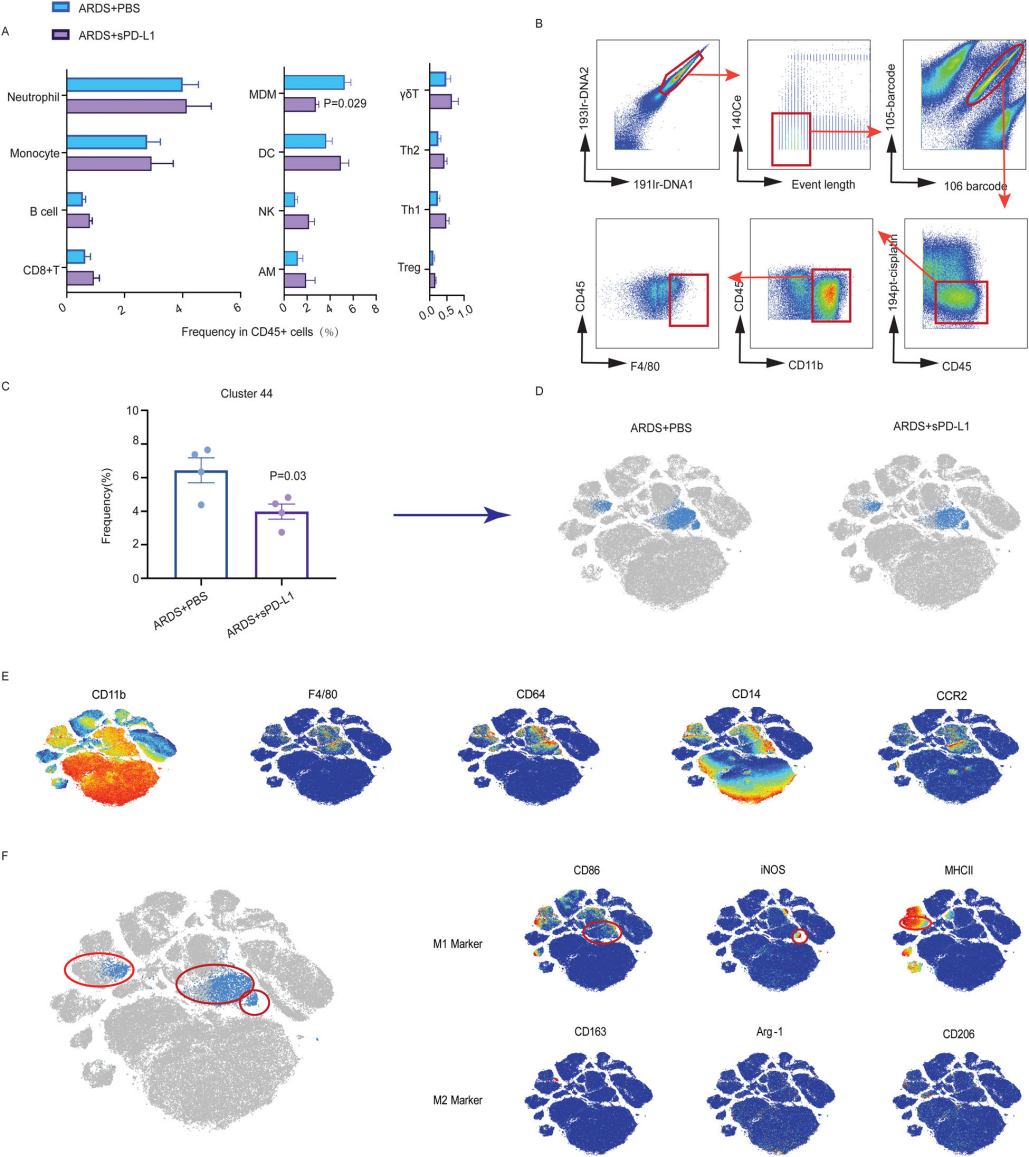
CyTOF revealed that administration of sPD-L1 reduced monocyte-derived macrophages. **A** The frequency of common immune cells in CD45+live cells by manual gating. **B** Gate strategy for monocyte-derived macrophages in one of the samples. **C** The frequency of significant reduced cluster in CD45+live cells (cluster 44). **D** The viSNE plot layered with the significant reduced cluster analyzed by X shift (*n* = 4/each group) (cluster 44). **E** The viSNE plots of expression of the markers that were highly expressed in the significantly changed clusters (cluster 44). The color gradient (from low (blue) to high (red)) indicates the intensity of the markers. **F** The viSNE plots of the markers of M1 like and M2 like macrophages in cluster 44. *P* value was analyzed by Man–Whitney test.

ARDS is characterized by the recruitment of excessive inflammatory cells, and the PD-1 pathway has been reported to induce apoptosis and attenuate immune cell proliferation^14^. Therefore, CyTOF was performed to screen for changes in common immune cells after the administration of sPD-L1. CyTOF was mainly focused on innate immune cells owing to the short interval of our observation (12 h). A total of 12 clusters-including Th1 (CD3 + CD4 + T-bet+), Th2 (CD3 + CD4 + Gata3+), CD8+ T cells (CD3 + CD8α+), γδT cell (CD3 + TCRγ+), Tregs (CD3 + CD25 + Foxp3+), B cells (CD3-CD19+), natural killer cells (CD3-NK1.1+), alveolar macrophages (CD11c + SiglectF+), dendritic cells (CD11c + MHCII+), neutrophils (CD3-Ly6G + CD11b+), monocytes (CD64-CD14 + CD11b + CD11c−) and MDMs (CD11b + F4/80+) were identified by manually gated of CD45+ live cells referred to the previous studies (Fig. 3A)^16,17^. We found a decrease of MDMs whereas other cells had no significant changes (Fig. 3A). Next, we used unsupervised methods to delineate the immune cell composition of lung, which can help to get unbiased results of the differences between two groups. A total of 49 clusters were identified by X-shift clustering analysis^18^. The heatmap in Fig. S3A shows the expression of multiple markers in all detected cell populations. The results of X-shift revealed two clusters (cluster 43 and 44) were significantly reduced in sPD-L1-treated ARDS mice compared to those in PBS-treated ARDS mice (Fig. 3C and Fig. S3B). We focused on one of the reduced clusters (cluster 44) because the other one accounted for a small proportion (<0.1%) and had no specific markers of immune cells (except for CD11b and Ki67). To better visualize the features of cluster 44 and further verify the results, we performed viSNE algorithm (20,000 cells/sample)^19^, each cell was represented as a dot, and the distance between the cells reflected the degree of similarity. Cluster 44 identified by X shift was layered to viSNE plot (Fig. 3D). It can be seen from the viSNE plots that the cell population had high expression of markers of both macrophages (CD11b, CD64, and F4/80) and monocytes (CD14 and CCR2), suggesting that this cluster was macrophages that recruited and developed from circulation monocytes (MDMs) (Fig. 3E).This was also consistent with the results of manual gating. Besides, the dispersed distribution of cluster 44 indicated its heterogeneity. We found this cell population consisted of three sorts of macrophages which all had high expression of M1-like macrophage markers (CD86, MHCII and iNOS) but almost no expression of M2-like macrophage markers (CD163, Arg-1 and CD206) (Fig. 3F), suggesting the proinflammatory property of this cell population.

**Fig. 4:**
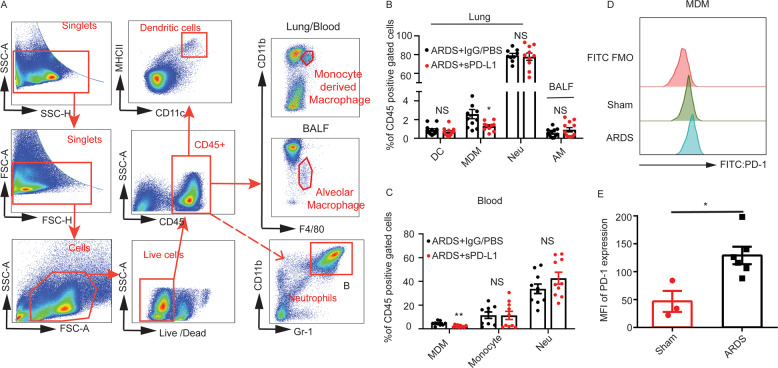
Flow cytometry verified that administration sPD-L1 reduced lung monocyte-derived macrophages. **A** Gate strategy for dendritic cells, monocyte-derived macrophages, neutrophils and alveolar macrophages (MDMs were measured in lung tissue single-cell suspension while alveolar macrophages were assayed in BALF) (*n* = 9–12/each group). **B** Percentage of different cell populations in CD45-positive cells of lung or BALF (*n* = 9–12/each group). **C** Percentage of different cell populations in CD45-positive cells of blood. **D** PD-1 expression on MDMs of lung (*n* = 9–12/each group). **E** Representative histograms of PD-1 expression on MDM of lung. The cells were previously gated by CD45^+^CD11b^+^F4/80^+^ (*n* = 3–6/each group). The experiments have been repeated for at least three times. Each value represents the mean ± SEM of three independent experiments. *P < 0.05, **P < 0.01, ***P < 0.001, analyzed by *t* test. MDM monocyte-derived macrophages, Neu, neutrophil.

To further verify the results and investigate the mechanism underlying the decrease in MDMs, we performed flow cytometry using collected bronchoalveolar lavage fluid cells, lung tissue single-cell suspensions and mice peripheral blood. The percentages of dendritic cells (CD11c^+^MHCII^+^), MDMs (CD11b^+^F4/80^+^), alveolar macrophages (CD11b^low^F4/80^+^), and neutrophils (CD11b^+^Gr-1^+^) in the lung tissue, BALF and peripheral blood were analyzed. MDMs (CD11b^+^F4/80^+^) were significantly reduced after the administration of sPD-L1, whereas the other cells were comparable between the two groups (Fig. 4A, B). We also assayed the MDMs (CD11b^+^F4/80^+^), monocytes (CD45^+^CD14^+^) and neutrophils (CD11b^+^Gr-1^+^) in blood. Consistent with that in the lungs, MDMs in blood significantly decreased after administration of sPD-L1 (Fig. 4C), whereas the monocytes showed no changes. Further, PD-1 expression in MDMs in lungs was upregulated in mice with direct ARDS, implying that sPD-L1 could engage with PD-1-expressing MDMs, thereby may reduce this cell population (Fig. 4D, E).

### The protective role of sPD-L1 was diminished in ARDS mice with monocytes/macrophages depletion

**Fig. 5:**
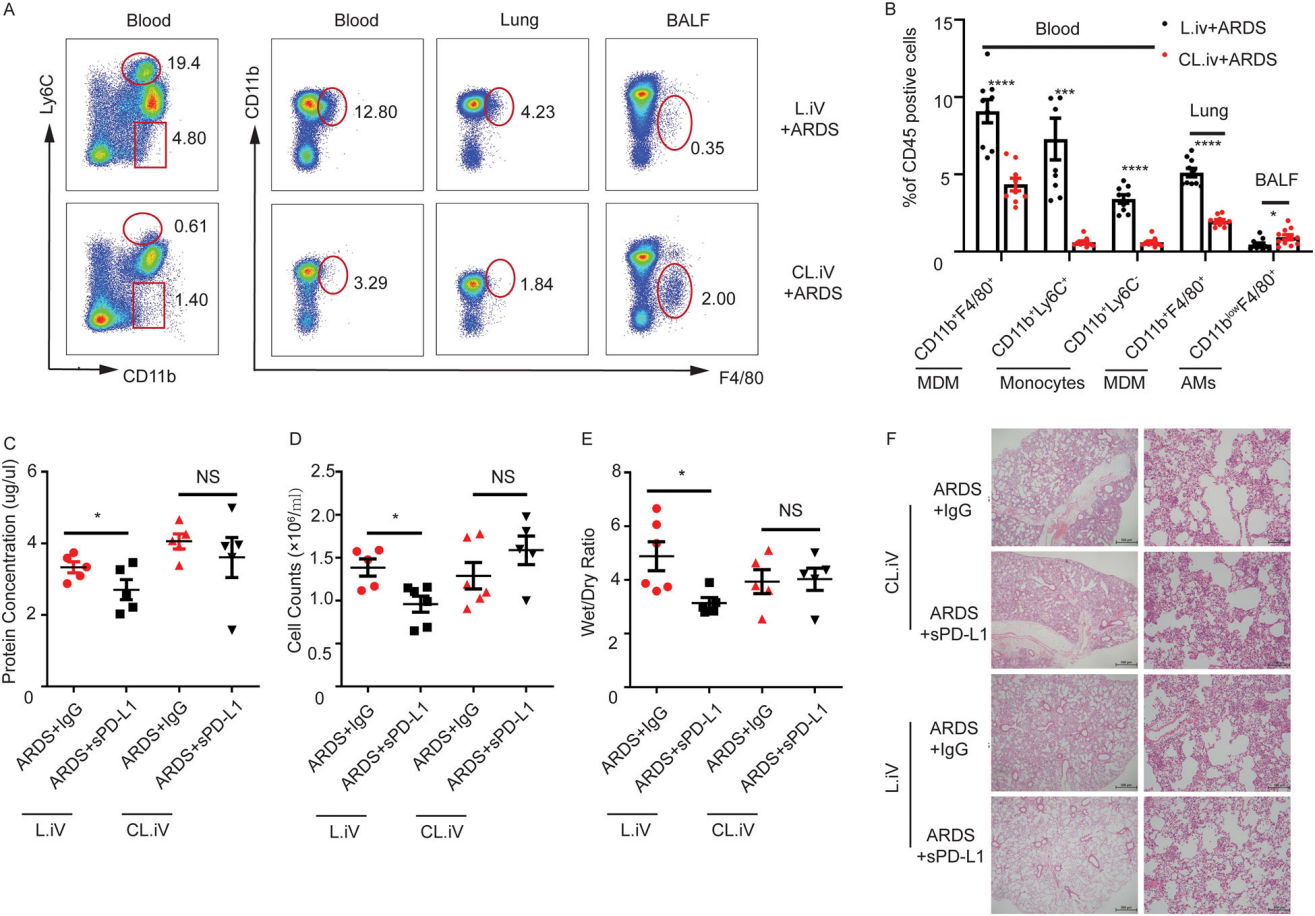
The protective role of sPD-L1 was diminished in ARDS mice with monocytes/macrophages depletion. **A** The flow cytometry of monocytes and macrophages in blood, lung and BALF of CL or L injected mice (*n* = 5/group). **B** The statistical graph of the frequency in CD45^+^cells of the cells in graph A (*n* = 5/group). **C** The protein concentration in BALF of mice with different treated methods (*n* = 5/group). **D** The cell counts in BALF of mice with different treated methods (n = 5–6/group). E) The wet to dry ratio of lung tissue of mice with different treated methods (*n* = 5–6/group). **F** The HE staining of lung tissue from different treated mice (*n* = 5–6/group). Each value represents the mean ± SEM of three independent experiments. The experiments have been repeated for at least three times. *P < 0.05, **P < 0.01, ***P < 0.001, analyzed by *t* test. Mo/Ma, monocyte/macrophage, MDM monocyte-derived macrophages, Neu, neutrophil.

To confirm that sPD-L1 reduced inflammatory lung injury by modulating MDMs, we depleted circulating monocytes by intravenously injecting clodronate liposome (CL). Over 90% of classical and non-classical monocytes (CD11b^+^ ly6C^+^ and CD11b^+^ Ly6C^-^) in blood were depleted (Fig. 5A), and the number of MDMs in blood was also decreased significantly. Approximately 50% MDMs in the lung were depleted (Fig. 5A, B). Next, we assayed the severity of ARDS in the Mo/Ma depleted mice. The protein concentration and white cell counts in BALF, as well as the wet/dry ratio of the lung were significantly decreased in the sPD-L1-treated mice in controls (Fig. 5C–E). However, this protective role of sPD-L1 was diminished in the Mo/Ma depleted mice (Fig. 5C–E). Likewise, HE staining showed the same results as for the other indicators (Fig. 5F).

### Administration of sPD-L1 induced apoptosis of MDMs

**Fig. 6:**
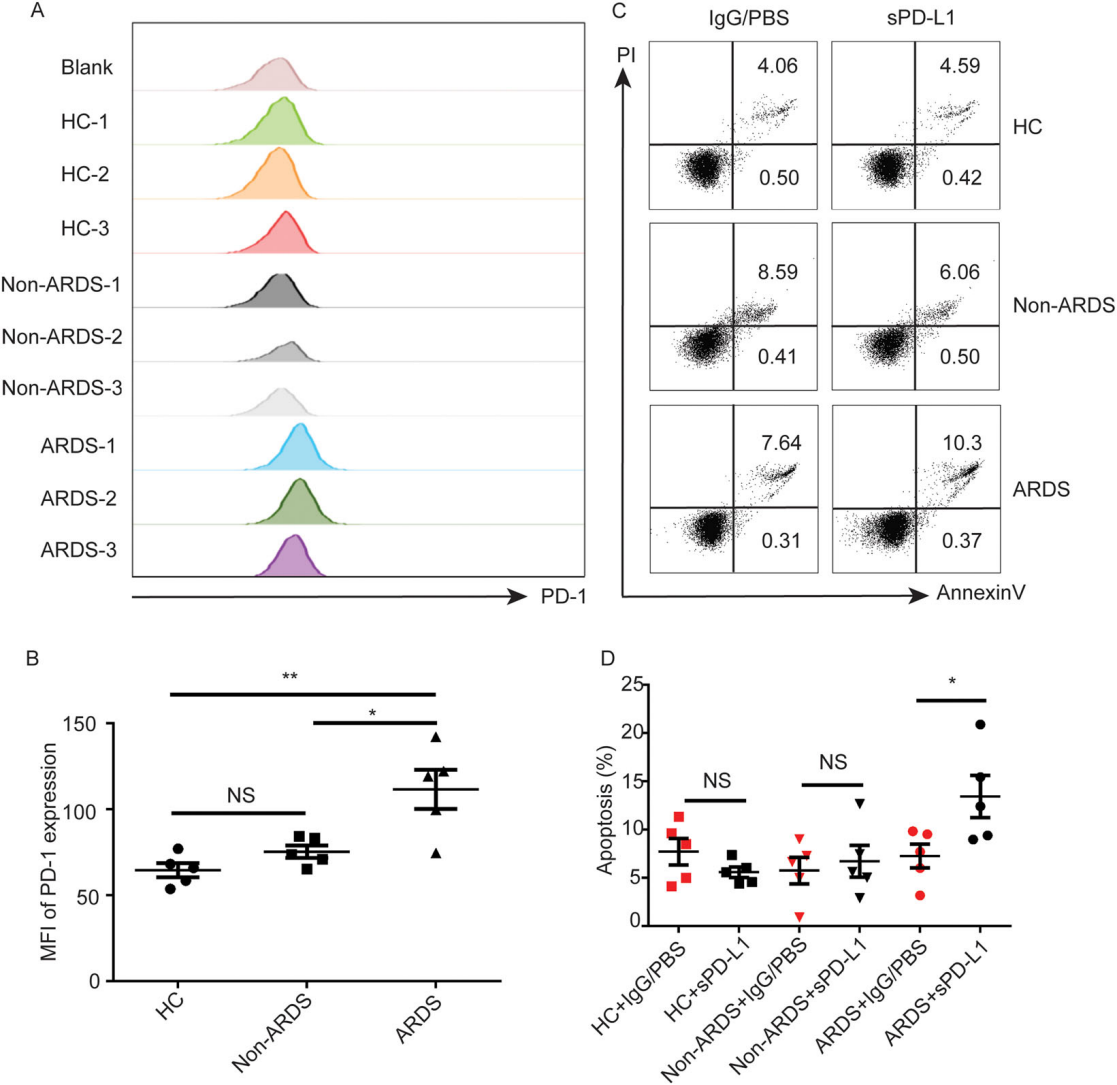
Administration of sPD-L1 induced apoptosis of monocyte-derived macrophages. **A** Representative histograms of PD-1 expression on monocyte-derived macrophages of healthy controls and ARDS patients. **B** PD-1 expression on MDM of healthy controls, Non-ARDS patients and ARDS patients. (*n* = 5/group) **C** The apoptosis of macrophages by PI/Annexin-V staining. **D** Apoptosis of macrophages in healthy controls, Non-ARDS patients and ARDS patients. (*n* = 5/group), analyzed by annexin V-fluorescein isothiocyanate/PI double staining. Cells in the B2 and B4 quadrants(annexin V + /PI+ and annexin V + /PI−, respectively) were considered to be apoptotic. **D** Graphical representation of apoptosis (*n* = 5/group). Each value represents the mean ± SEM of three independent experiments. *P < 0.05, **P < 0.01, ***P < 0.001, analyzed by *t* test. MDM monocyte-derived macrophages; HC healthy controls.

The quantity of MDMs was determined based on different factors, mainly including the apoptosis and proliferation of the MDMs and the recruitment of circulating monocytes. Our results showed that the proliferation marker Ki67 in MDMs did not differ significantly between the two groups. In terms of recruitment, no change was found in the circulating monocytes and serum monocyte chemoattractant protein-1 (MCP-1) (Fig. S4). Thus, we hypothesized that sPD-L1 induced the apoptosis of MDM_S_. To verify this, the apoptosis of MDMs from mice were assayed. Peritoneal macrophages induced by thioglycolate medium were derived from monocytes in circulation^20^, thus were used to determine the apoptosis of MDMs induced by sPD-L1. We found that peritoneal macrophages treated with sPD-L1 had higher percent of apoptosis cells compared to IgG-treated ones (Fig. S7). To further confirm this result in human MDMs, blood from ARDS patients, non-ARDS patients, and healthy controls was collected (Table 2). Monocytes were isolated and differentiated into macrophages. Compared to that in non-ARDS patients and healthy controls, in the ARDS patients, PD-1 expression in MDMs increased (Fig. 6A, B). We found a significant increase in the apoptosis of MDMs obtained from ARDS patients in sPD-L1-treated group compared to IgG/PBS-treated group, whereas the effect didn’t show in those of controls (healthy individuals and non-ARDS patients) (Fig. 6C, D). Therefore, these results implied that the engagement of sPD-L1 on PD-1-expressing macrophages induced the apoptosis of MDMs.

**Table 2:** Demographics of patients whose blood was collected for experiments in vivo.

	ARDS (*n* = 5)	Non-ARDS (*n* = 5)	Healthy controls (*n* = 5)
Age	61 ± 25	64.8 ± 6	26.0 ± 2
Female	3/5	3/5	4/5

## Discussion

Current treatments for ARDS mainly depend on supportive methods such as lung-protective ventilation. In the last decade, trials of novel drug therapies for improving ARDS outcomes have emerged, but none showed efficacy in phase II and III trials^21–26^. The lack of specific and effective pharmacotherapies for ARDS contributes to the high mortality of ARDS^26^. Our study is the first to demonstrate that sPD-L1 was associated with the mortality in patients with direct ARDS and that it played protective roles in the corresponding experimental models. It may lead to the establishment of a new therapeutic modality for a specific subset of ARDS to manipulate the inhibitory function of PD-1 by sPD-L1. Furthermore, CyTOF revealed that sPD-L1 reduced a specific cluster of MDMs in the lung and that Mo/Ma depletion diminished the effects of sPD-L1 on lung inflammation and injury. sPD-L1 induced the apoptosis of MDMs in ARDS patients but not in non-ARDS patients or healthy controls, which may be related to the high PD-1 expression in MDMs in ARDS patients. Our findings suggest that sPD-L1 is a promising agent in the treatment of patients with direct ARDS in clinical practice, and it likely acts by targeting MDMs.

Patients with ARDS can be subclassified as having direct (pulmonary) or indirect (extrapulmonary) ARDS^27^. There exist significant differences between direct and indirect ARDS in terms of gene sets, metabolic profiles, pathophysiology, clinical outcomes, and response to drug treatment^27–29^. Our results revealed that in direct ARDS, serum sPD-L1 was significantly increased in survivors than in non-survivors, but not in the whole cohort of ARDS. Compared to that reported previously, distinct effects of PD-1 activation were found between the two subsets of ARDS. Our study focused on direct ARDS, whereas Joanne et al. investigated the PD-1 pathway in mice with sepsis induce indirect ARDS. In the contrast to our findings, they demonstrated that *PD-1/PD-L1* gene deficiency imparted survival benefits in indirect ARDS. One reason for this could be the different roles of inflammatory responses in these two subsets. Morrell et al. found that some important inflammatory response gene sets were significantly associated with survival and ventilator-related outcomes in direct ARDS, while these results were not observed in indirect ARDS^28^. Therefore, patients with direct ARDS were more likely to benefit from the regulation of inflammatory responses by the PD-1 pathway. In our study, high levels of sPD-L1 predicted better prognosis for direct ARDS. However, high levels of sPD-L1 were reported to be associated with high mortality in sepsis patients^30^, probably owing to defects in immune functions of patients caused by the activation of PD-1 pathway^30,31^. These results implied that the immunosuppressive activity of sPD-L1 may lead to different, or even opposite outcomes in various subsets or diseases depending on their responses to immune inhibition.

Accumulated evidence has demonstrated that high levels of sPD-L1 are associated with immunosuppression^32–35^. Consistent with this, our results showed that the administration of sPD-L1 reduced inflammatory lung injury in mice with direct ARDS, which may be attributed to the decrease of proinflammatory macrophages. Immune cells recruitment is involved in inflammatory lung injury through an array of complex mechanisms and drive the development of ARDS^26^. Macrophages participate either in the induction or resolution of ARDS depending on their distinct functional phenotypes^36–38^. Classically, activated (M1) macrophages facilitate host defense and become more proinflammatory while the alternatively activated (M2) macrophages maintain homeostasis and possess anti-inflammatory properties. Our results showed that the decreased MDMs had high expressions of markers in M1-like macrophages, suggesting that these promoted inflammation in ARDS. Other studies have also reported that MDMs were recruited into the lung when infection occurred^39,40^ and were thought to be one of the main effectors of inflammatory lung injury^41^. sPD-L1 reduced the number of these macrophages and may help control the overwhelming immune responses and reduce inflammatory lung injury.

Macrophages have been widely described as pivotal factors in the development of ARDS while the roles of T cells are remain unclear^38,40,41^. However, giving that the activation of PD-1 pathway is well-known for its role in reducing the functions and/or inducing apoptosis of T cells^14^, we also detected changes in T cells using CyTOF after the administration of sPD-L1. We didn’t find any significant changes in the amounts of T cells, including Th1(only a trend of increase), Th2, Tregs, and CD8+ T cells and their corresponding cytokines secretion. The main possible reason for this may be the short disease course in our murine model. The differences in survival rates between the sPD-L1-treated and IgG-treated groups can be observed early enough (18–24 h), and the alleviation of inflammatory lung injury upon the administration of sPD-L1 may occur much earlier (12 h). During this period, innate immune cells were rapidly activated and recruited into the lung tissue and airways (within 24 h) whereas adaptive immune cells take about 72 h to be recruited to the infection sites through peripheral circulation after proliferation and differentiation in secondary lymph nodes^42,43^. Although sPD-L1 was able to locate in lung, it might have exerted its functions mainly in circulating cells rather than in lung resident cells because it was administrated by intravenous injection. Therefore, we only found a decrease in MDMs but not in other lung resident cells, and the quantity of the circulating MDMs was also observed after the administration of sPD-L1. Nevertheless, whether serum sPD-L1 regulated T cells in ARDS over a longer interval or by other methods of administration (intratracheal injection) was not conclusive in our study. In addition, the contribution of sPD-L1 in inhibiting the cytokine storm of T cells may be more important in ARDS induced by virus than bacteria, which was not involved in the present study^44,45^. It will be interesting to explore the role of sPD-L1 in virus-induced ARDS in further studies. The role of sPD-L1 in innate immune and adaptive immune responses and the consequent results in lung injury can be extremely complex in the human body. However, our study, provided supportive evidence on the protective role of sPD-L1 in direct ARDS, which can be partially explained by the reduction of MDMs.

The regulatory effects of PD-1 signaling in macrophages have been increasingly studied as they have been found to participate in a wide range of diseases^6,46–48^. In a recent study, macrophages obtained from patients with ARDS were subjected to transcriptomic analysis. The results showed that PD-1/PD-L1 pathway-associated gene sets were significantly decreased in macrophages obtained from patients who underwent prolonged mechanical ventilation or died^28^. These results suggested that the activation of PD-1 pathway in macrophages might exert protective effects in patients with ARDS. We found the expression of PD-1 in MDMs increased in the mice with direct ARDS than in the controls. Consistent with the findings in mice, we also observed a significant increase of PD-1 expression in MDMs from ARDS patients compared to in those from non-ARDS patients and healthy controls. The increased expression of PD-1 in MDMs provided more targets for sPD-L1, which may increase the possibility of PD-1 activation by binding to sPD-L1. PD-1 was reportedly involved in macrophage apoptosis^49^. Further, sPD-L1 induced the apoptosis of MDMs in direct ARDS. This could contribute to the reduction of macrophages. However, we acknowledge that other mechanisms may also be involved.

There were several limitations to the present study. The limited number of patients with direct ARDS due to the relatively low morbidity was the main limitation. Second, we investigated the levels of sPD-L1 only in the serum, which indirectly represents the lung immune environment. Additional studies are needed to investigate the origin of sPD-L1 and the association between sPD-L1 in the serum and BALF. However, serum can be easily sampled and may be better biomarker for the prognosis of direct ARDS. Third, the APACHEII score was not controlled for in ARDS and non-ARDS patients because we had avoided including the patients with diseases that were reported to be associated with sPD-L1 in control group, for instance, sepsis and pancreatitis. Fourth, we only investigated the protective role in pseudomonas aeruginosa induced ARDS model, giving the biological variability of bacteria, it need to be cautious to over interpret our results. Last, the apoptosis of MDMs induced by sPD-L1 in vitro only revealed a potential mechanism of the decrease in lung MDMs.

**Fig. 7:**
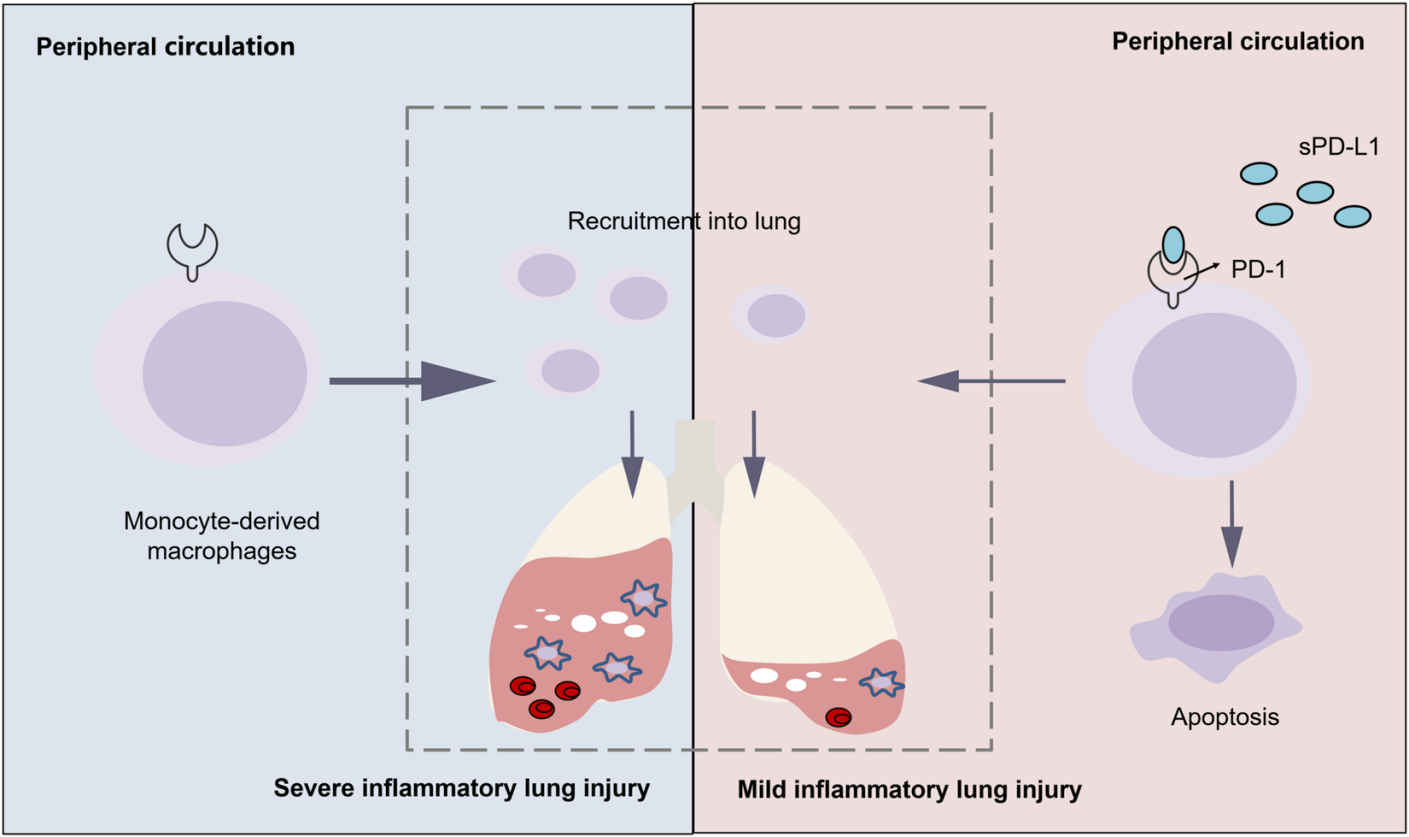
A schematic diagram explaining the role of sPD-L1 in direct ARDS. In the setting of direct ARDS, soluble PD-L1 binded to the monocytederived dmacrophages with increased PD-1 expression and induced their apoptosis. The reduced MDMs alleviated the inflammatory lung injury and ultimately improved direct ARDS.

In conclusion, this is the first study to show that elevated serum sPD-L1 levels had a protective role in direct ARDS. Mouse experiments further revealed that sPD-L1 relieved inflammatory lung injury by reducing the proinflammatory MDMs (CD11b^+^ F4/80^+^), the underlying mechanism was related to the apoptosis of MDMs (Fig. 7). sPD-L1 may be a potential drug target as well as a useful biomarker for predicting the prognosis of direct ARDS.

## Materials and methods

### Human study subjects

A total of 43 patients diagnosed with ARDS, 20 non-ARDS patients receiving mechanical ventilation at the ICU of Ruijin Hospital, China and 10 healthy controls from the Healthy Center of the Ruijin Hospital were enrolled in this study. The inclusion criteria of ARDS were in accordance with the Berlin definition^50^. Patients with lung cancer, autoimmune disease, or long-term glucocorticoid administration were excluded. Blood samples were collected for sPD-L1 enzyme-linked immunosorbent assay within 48 h of ARDS onset. All human subjects provided written informed consent before enrollment into the study. On the same day, demographic characteristics, Acute Physiology and Chronic Health Evaluation (APACHEII) score, oxygenation index (PaO_2_/FiO_2_), and laboratory test results (white cell counts, neutrophil counts, lymphocyte counts, C-reactive protein, procalcitonin) were collected. The patients were followed until death or discharge. The primary outcomes were ICU mortality, while secondary outcomes included ventilator-free days and ICU stay. The study was approved by the Ruijin Hospital Ethics Committee Shanghai Jiao Tong University School of Medicine. This trial is registered with the Chinese Clinical Trial Registry under number ChiCTR1800015930. Registered April 29, 2018, http://www.chictr.org.cn/edit.aspx?pid=25609&htm=4.

### ELISA

sPD-L1, sPD-1, tumor necrosis factor (TNF)-α, interleukin (IL)-17, and IL-10 were measured in enzyme-linked immunosorbent assays according to the manufacturer’s instructions (Human TNF- α ELISA Kit, EL10019, Anogen, Ontario, Canada; Human IL-10 ELISA Kit, EL10027, Anogen; Human IL-17A ELISA Kit, EL10053, Anogen; Human/Cynomolgus Monkey B7-H1/PD-L1 Immunoassay, DB7H10, R&D Systems, Minneapolis, MN, USA; Human PD-1 ELISA Kit, EL10059, Anogen). Each sample was analyzed in duplicate. The intra-assay and inter-assay coefficients of variation were below 20%. The minimum detectable concentration was 5 pg/mL for human IL-17A, 3.5 pg/mL for human IL-10, 0.369 pg/mL for human PD-L1,0.44 pg/mL for human PD-1 and 2.4 pg/mL for human TNF-α.

### Animal procedures

The animal study was approved by the University Committee for Laboratory Animals and was performed in accordance with the guidelines of the Shanghai Institutes for Biological Sciences Council on Animal Care. Male C57BL/6 mice (8–10 weeks old) were intratracheally injected with *Pseudomonas aeruginosa* [PAO1, 2 × 10^6^ colony-forming units (CFU), ATCC, Manassas, VA, USA] in 50 µL PBS or PBS as a control. After 6 h, mice were randomly allocated to experimental or control groups, after that, 20 µg PD-L1-Fc fused protein (Recombinant Mouse B7-H1 (PD-L1, CD274)-Fc Chimera (carrier-free), Biolegend, San Diego, CA, USA) in 50 µL PBS or an equivalent amount of PBS was administrated by intravenous injection. The mice were sacrificed at 12 h post-infection. The experiments for verifying the protective role of sPD-L1 in mice were repeated for more than three times and the investigator was blinded to the group allocation and assessing the outcome during one of the experiments.

### Detection of the distribution of sPD-L1 in lung

The mice were intratracheally injected with *Pseudomonas aeruginosa*. After 6 h, 20 µg sPD-L1-His fused protein (PD-L1 Protein, Mouse, Recombinant (His Tag), 50010-M08H, SinoBiological) was injected into C57BL/6 mice intravenously. The mice were then sacrificed at 30 min, 1 h, and 2 h post injection to obtain the lung. Lungs were disected and lyzed with RAPI buffer (NCM biotech, China) containing compete EDTA-free protease inhibitor (Roche) and 1 mM PMSF (Amresco) on ice for 1 h. Twenty-five micrograms of soluble protein was separated by 10% SDS-PAGE and immunoblotted with mouse anti-his-Tag antibody (Proteintech, Catalog number: 66005-1-Ig,CloneNo.: 1B7G5). The immunohistochemistry of lung was performed as previously reported^51^. In brief, the lung was paraffin embedded and dewaxed, then citrate buffer was used for antigen unmasking. After incubation with His antibody at 4 °C (Mouse monoclonal [HIS.H8] to 6X His tag, Abcam, ab18184) overnight, the lung sections were incubated with horseradish peroxidase (HRP) for 30 min.

### Assessment of lung injury

BALF was attained as reported previously^52^. In brief, the lungs were perfused with 1.5 mL of PBS (3 times, 0.5 mL/perfusion) using a 20-gauge endotracheal catheter, followed by the collection of BALF from the right lung (the left lung was ligated with string). BALF samples were centrifuged at 500 × *g* for 5 min, and then the supernatant was used to assess the protein concentration by bovine serum albumin protein assay (Sigma-Aldrich, St. Louis, MO, USA) and the pellet was treated with red blood cell lysis buffer (ACK Lysis Buffer, Gibco, Grand Island, NY, USA) to remove the red cells and then assayed for white cell counts with a cell counter (Jimbio, Jimbio Technology, Jiangsu, China). The left lung of the mice was processed for hematoxylin and eosin (HE) staining and the wet and dry lung weights were determined to calculate the wet/dry ratio of the lung. The levels of TNF-a in BALF was determined by ELISA kit (Mouse TNF- α ELISA Kit, MEC1003, Anogen).

### Bacterial load in the bronchoalveolar lavage fluid

The bacterial load in the bronchoalveolar lavage fluid was determined as we previously described^53^.

In brief, the BALF were collected and diluted into PBS. After that, 50 µl diluent were plated on LB agar containing ampicillin and incubated at 37 °C overnight for the growth of pseudomonas aeruginosa. The limit of detection was 1 CFU in 100 µl of BALF.

### Mass cytometry

The lung was obtained from the mice with ARDS treated with sPD-L1 (*n* = 4) or PBS (*n* = 4). Lung tissues were cut into small pieces and digested with 50 µL of 0.1 mg/mL collagenase I (Collagenase, Type 1, Sangon Biotech, Shanghai, China) at 37 °C for 1 h to prepare a single-cell suspension. Samples for CyTOF were prepared as the previous reports^18^. To stimulate the cells, 2 μL cell of cell activation cocktail (BioLegend, 423303) was added, and the cells were then incubated for 4 h. Next, a single-cell suspension was labeled using 194Pt at 4 °C for 5 min to distinguish between live and dead cells. Further, 50 μl of Fc blocking mix was added for 20 min to block the FcR-involved unwanted staining. The cells were incubated for 30 min at 4 °C in 100 µL of DPBS/0.1% BSA containing antibody cocktail prepared with 41 antibodies (listed in Supplementary Table S1) purchased from BioLegend, eBioscience™, BioRAD, BD, BioXcell and R&D. Metal tags were linked using the Maxpar X8 Antibody Labeling Kit (Fluidigm). The cells were then fixed overnight with 1 mL Fix&Perm Buffer (Fluidigm) containing 1 µL of DNA Intercalator-Ir (Fluidigm) to discriminate of dead cells from live cells or to discriminate single nucleated cells from doublets. The following day, the cells were incubated for 30 min at room temperature in 1 mL Nuclear Antigen Staining Buffer (Fluidigm). Intracellular or nucleus antibody mix was added to 50 µL of Nuclear Antigen Staining Perm (Fluidigm) and incubated for 30 min. The sample was barcoded by a unique barcode isotope combination before the run on CyTOF. CyTOF assay was performed on a Helios 2.0 mass cytometer (Fluidigm) according to the manufacturer’s instructions at an events rate of 300 events/s. Events were first normalized by bead normalization and debarcoded to obtain the data of each sample. The data were then manually gated in FlowJo V10 to remove debris, dead cells, doublets and non-immune cells (CD45 negative cells) prior to analysis.

### Data processing

The data were analyzed as previously described^54^. The supervised analysis was first performed to detect the frequency of common immune cells in CD45+ live cells by manual gating. Mann–Whitney *U*-tests were used to compare the differences of these cell populations in ARDS mice treated with sPD-L1 or PBS. After that, live CD45+ cells identified by manually gating were further analyzed by two unsupervised methods (X-shift clustering and viSNE algorithms)in MATLAB R2018a to delineate the immune cell composition and detect specific cell populations. ViSNE (20,000 cells/ sample) is a dimensionality reduction algorithm that distributes cells in a 2D dot plot wherein each cell is represented as a dot, with two parameters were set: t-distributed stochastic neighbor embedding 1 (tSNE1) and tSNE2. The distance between the cells in viSNE reflects the degree of similarity^15^. X-shift clustering uses weighted *k*-nearest-neighbor density estimation. The data were performed arcsinh switch (cofactor = 5) before the clustering analysis. A total of 22 clusters were identified by X shift and annotated manually according to the markers for different cell populations^54^. The heatmap of clusters was normalized by setting the 1st percentile as the minimum value and the 99th percentile as the maximum value.

### Flow cytometry

The lung tissue from mice was cut into small pieces and digested with 50 µL 0.1 mg/mL collagenase I (Collagenase, Type 1, Sangon Biotech, Shanghai, China) at 37 °C for 1 h to prepare a single-cell suspension. The cells collected from the BALF and lung single-cell suspension were used for flow cytometry. The following fluorescent antibodies were purchased from Biolegend: allophycocyanin (APC)/Cy7 anti-mouse CD11c,117323; Brilliant Violet 421 anti-mouse F4/80, 123131; PerCP-Cy™5.5 rat anti-mouse I-A/I-E,107626; APC anti-mouse CD274 (B7-H1, PD-L1),124311; Fluorescein isothiocyanate anti-mouse CD279 (PD-1),135213; Brilliant Violet 421™ anti-mouse CD3,100228; APC anti-mouse CD4,100412; Phycoerythrin/Cy7 anti-mouse/human CD11b,101216; Dead/live staining (LIVE/DEAD™ Fixable Aqua Dead Cell Stain Kit) from Invitrogen (Carlsbad, CA, USA) was used to remove dead cells and debris. The Fluorescence Minus One (*FMO controls*) were used to set the gates.

### Monocyte/macrophage depletion

For depletion of circulating monocytes, mice were intravenously injected with 200 μL of CL intravenously 12 h before the ARDS model was established. The same volume of liposome (L) was injected into the control mice. sPD-L1 or IgG/PBS was injected 6 h after intratracheally injection of PAO1. Simultaneously, 100 μL of CL or L was injected as a sustained dose to deplete Mo/Ma. Twelve hours later, the BALF, circulating blood, and lung tissue were obtained for subsequent experiments.

### Peritoneal macrophage collection and treatment

The peritoneal macrophages were collected as described before^55^. In brief, 1.5 ml thioglycollate medium was injected into the peritoneum of C57BL/6 mice. After 4 days, the mice were sacrificed and 8.5 ml ice-based RPMI medium containing 10% bovine serum was injected into the peritoneum of mice. To harvest the peritoneal cells, the mice were shaken for several times and the peritoneal lavage were then extracted by syringe. The collected cells were centrifuged and treated with red cell lysis buffer for 10 min. The remaining cells were immediately seeded into 6-well plate at 37 °C. To purify the macrophages, the non-adherent cells in cell culture medium were discarded 2 h later, and the cells were washed for three times using PBS. The peritoneal macrophages were then pretreated with 10 µg/mL sPD-L1 or IgG 12 h before subjected to the LPS (10 µg/mL) stimulation. After 12 h, the apoptosis of these cells were determined by flow cytometry.

### Culture human MDMs

Peripheral blood mononuclear cell (PBMC) were obtained from healthy controls, Non-ARDS patients with mechanical ventilation, and ARDS patients using Ficoll-Paque (Ficoll-Paque PREMIUM,GE) Gradient extraction method. The PBMCs were transferred into 10 cm^2^ cell culture plates. Nonadherent cells were removed after incubation in a 37 °C cell culture incubator with 5% CO_2_/95% air for 30 min. Adherent cells were considered as MDM. Next, 20 ng/mL of GM-CSF (Recombinant Human GM-CSF,AF-300-03,Peprotech) was added to the MDMs, and the culture medium and GM-CSF was replaced every 2 d. Macrophages were obtained after 7 d and verified by flow cytometry.

### Assay the apoptosis of macrophages

The macrophages were stimulated with 100 ng/mL of LPS (sigma) for 12 h and co-incubated with 5 μg/mL PD-L1-Fc protein (Recombinant Human B7-H1 (PD-L1, CD274)-Fc Chimera (carrier-free), BioLegend) or human IgG (Sigma) for 6 h. Apoptosis was analyzed by PI/Annexin-V staining (Annexin V-FITC/PI Apoptosis Detection Kit, 40302ES20, YEASEN) according to the manufacturer’s instructions.

### Statistical analysis

Experiments were repeated using different donors and animals, respectively, in at least triplicates. The data were analyzed using SPSS 19.0 software (SPSS, Inc., Chicago, IL, USA) and descriptive statistics are presented as the mean and standard deviation for continuous variables and absolute and relative frequencies for categorical variables. Power calculations were performed before experiments were conducted to determine appropriate sample size. The data shown in graph are presented as the mean ± SEM. All the data were tested for the normal distribution by Kolmogorov–Smirnov test. Except for the levels of sPD-L1 in non-ARDS patients, levels of sPD-1, IL-10, IL-17 in ARDS patients and normal controls, other indicators and factors were normally distributed. Student *t* test was used to compare continuous normally distributed variables, while Mann–Whitney *U*-tests were for continuous non-parametric variables. Categorical data were tested using the chi-squared test (or Fisher’s exact test, if necessary). Correlations were assessed by Pearson test, and *P* < 0.05 was considered to indicate statistical significance.

## Supplementary information

FigureS1

FigureS2

FigureS3

FigureS4

FigureS5

FigureS6

FigureS7

Supplementary table1

Supplementary table2

Supplementary figure legend
