# Supplementary figures and images for: Soluble PD-L1 improved direct ARDS by reducing monocyte-derived macrophages
Source: Cell Death Dis. 2020 Oct 30;11(10):934. doi: 10.1038/s41419-020-03139-9 (PMC7596316; doi:10.1038/s41419-020-03139-9)

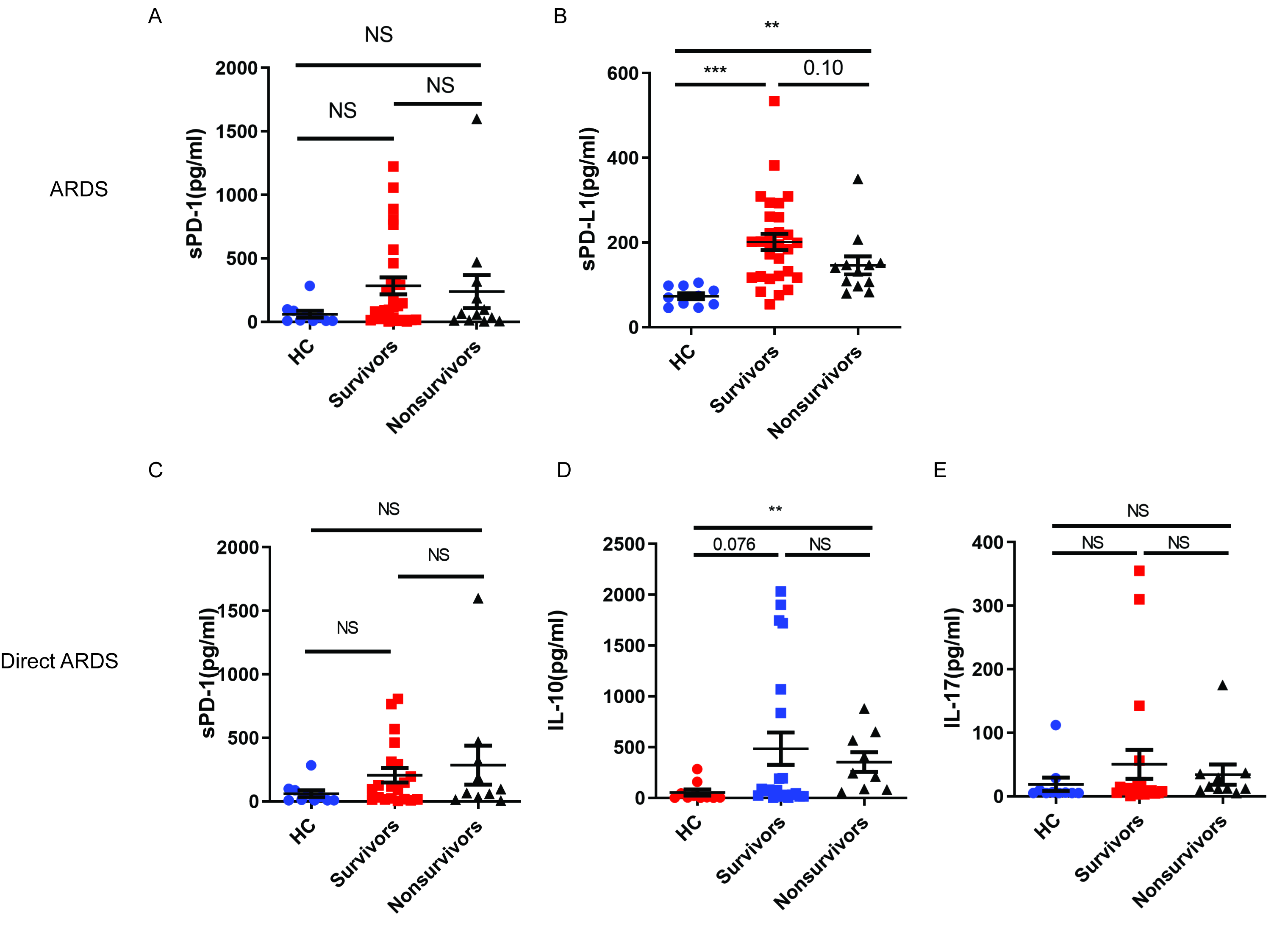

Supplement: Supplementary file 1 — FigureS1 [file 41419_2020_3139_MOESM1_ESM.tif]

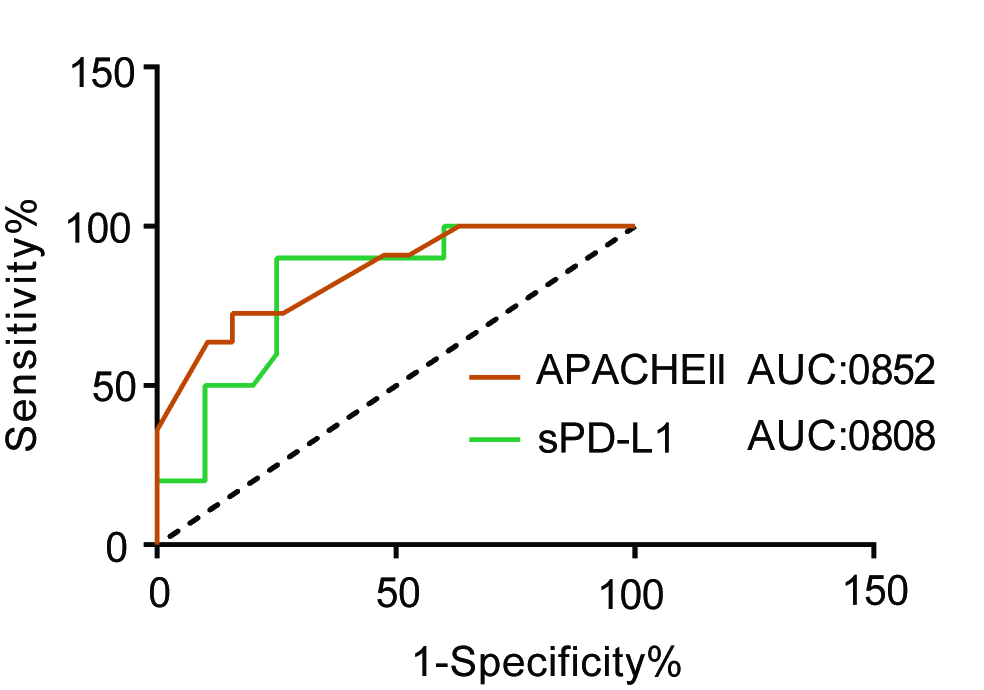

Supplement: Supplementary file 2 — FigureS2 [file 41419_2020_3139_MOESM2_ESM.tif]

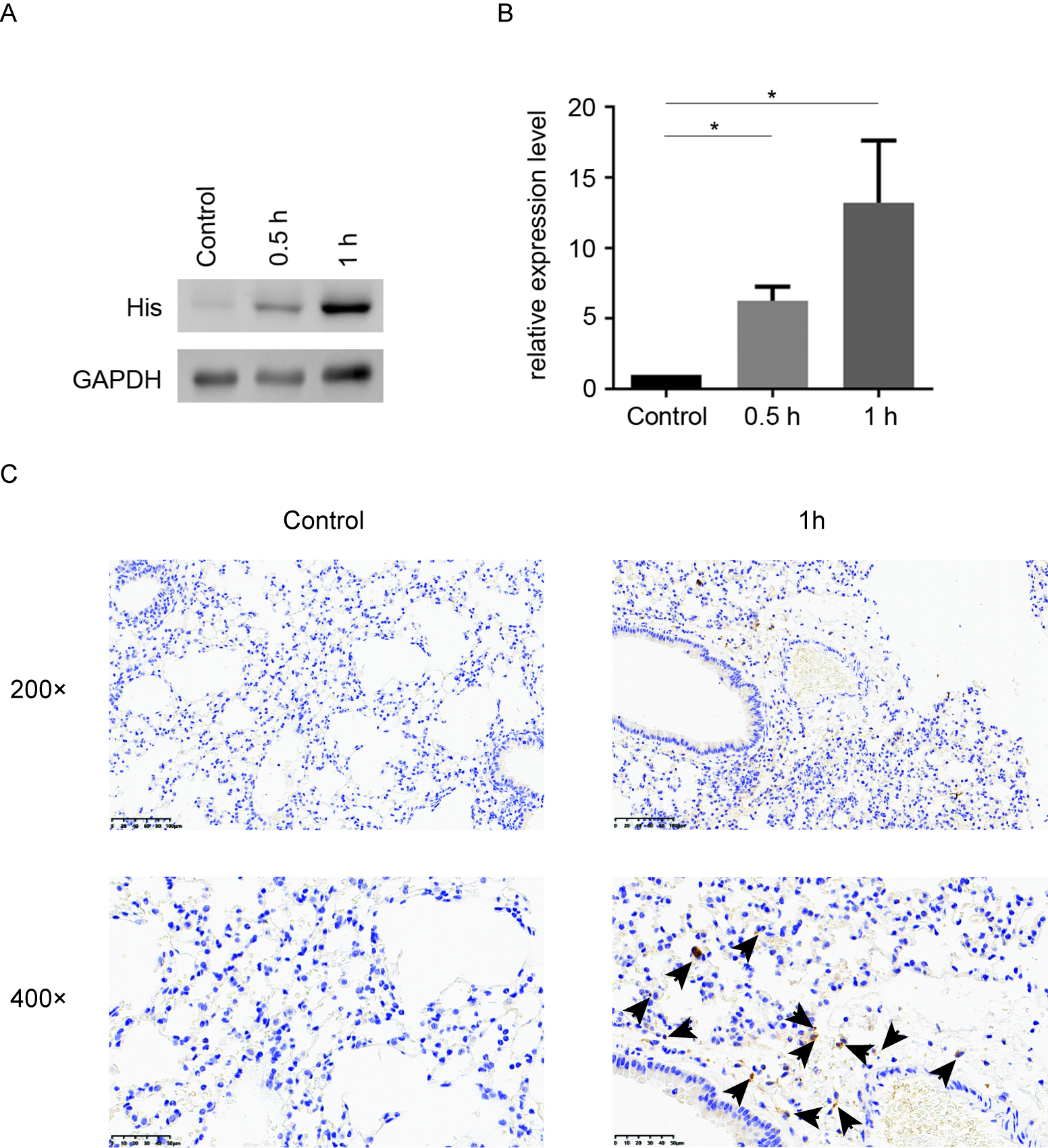

Supplement: Supplementary file 4 — FigureS4 [file 41419_2020_3139_MOESM4_ESM.tif]

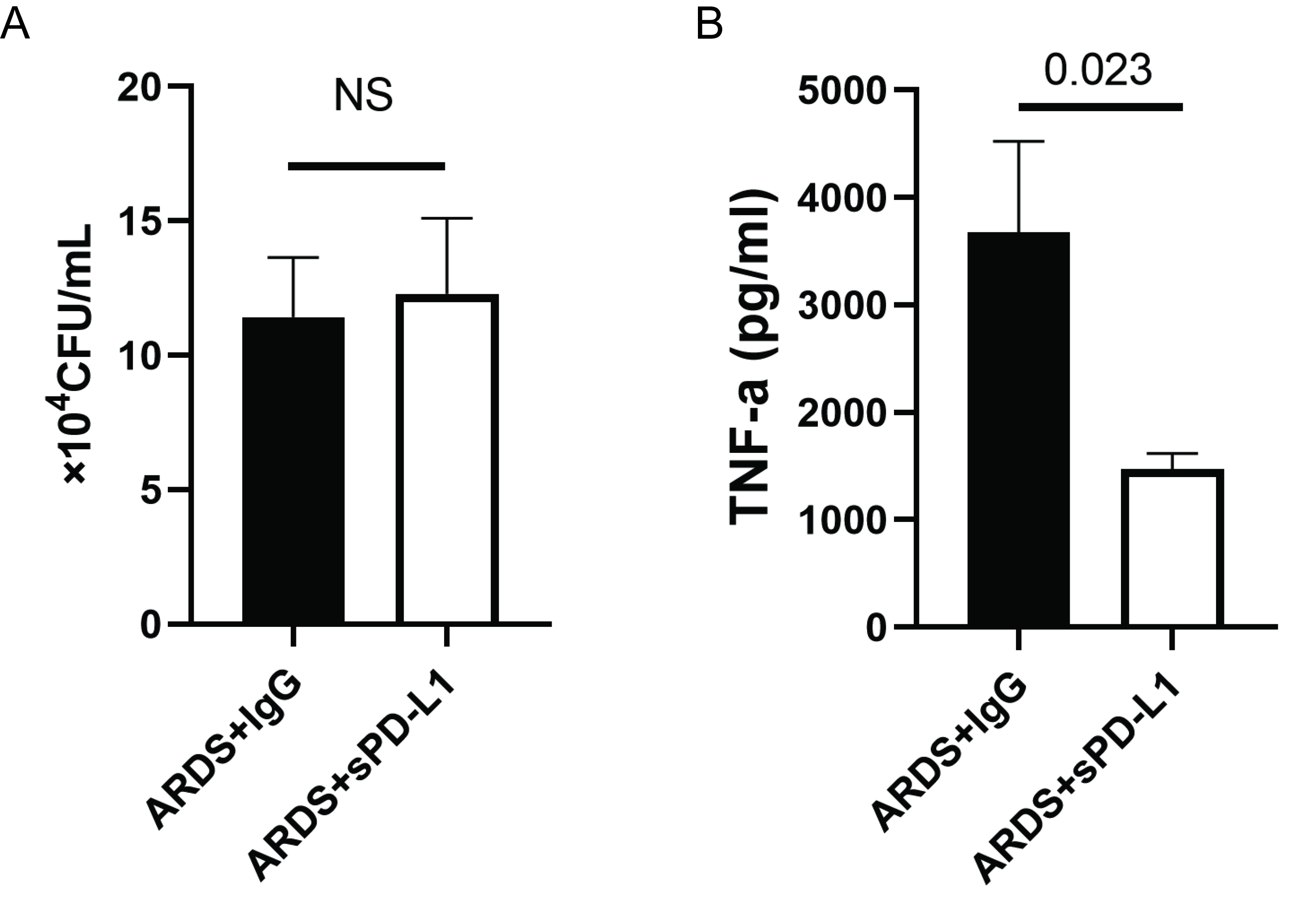

Supplement: Supplementary file 5 — FigureS5 [file 41419_2020_3139_MOESM5_ESM.tif]

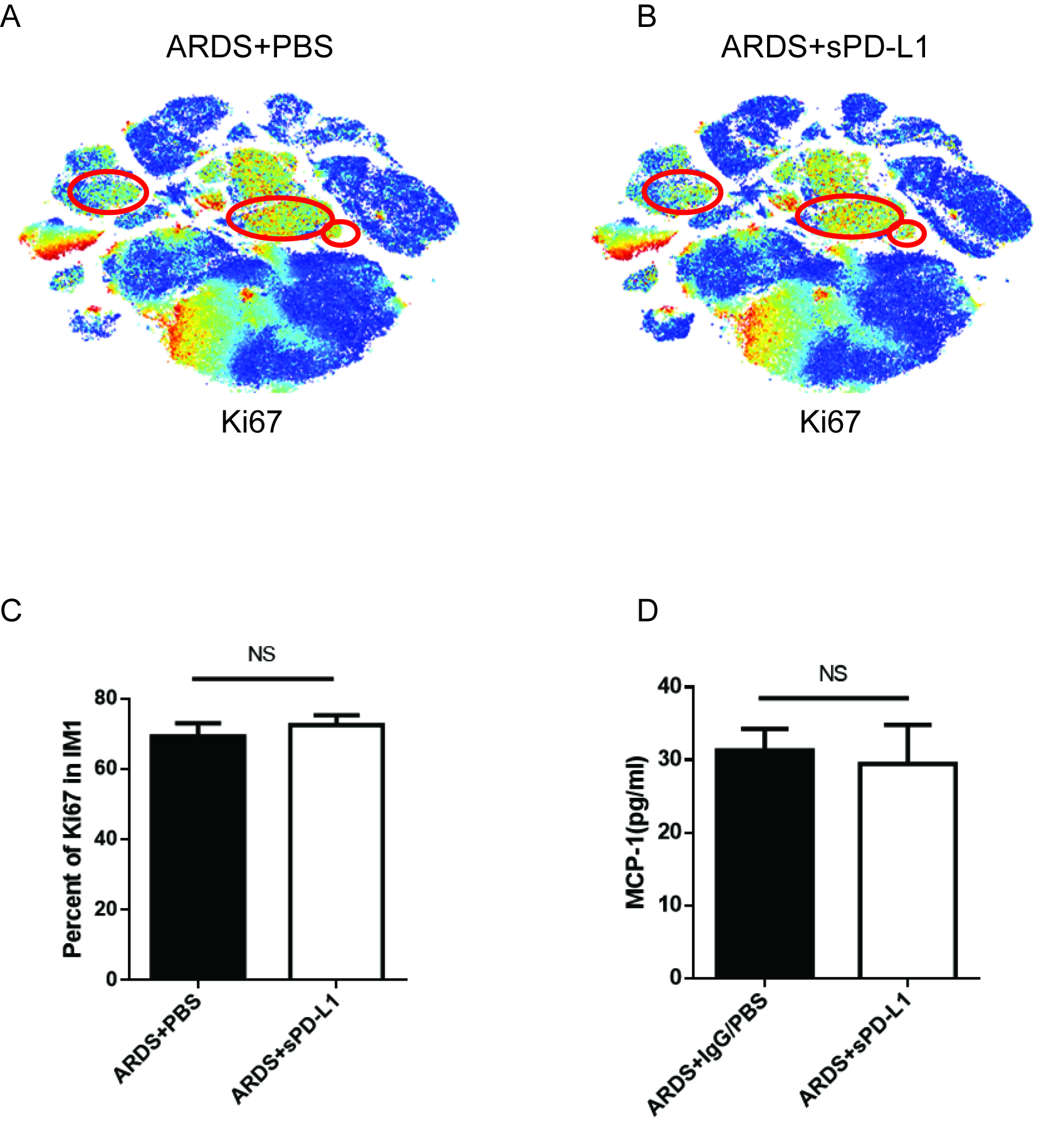

Supplement: Supplementary file 6 — FigureS6 [file 41419_2020_3139_MOESM6_ESM.tif]

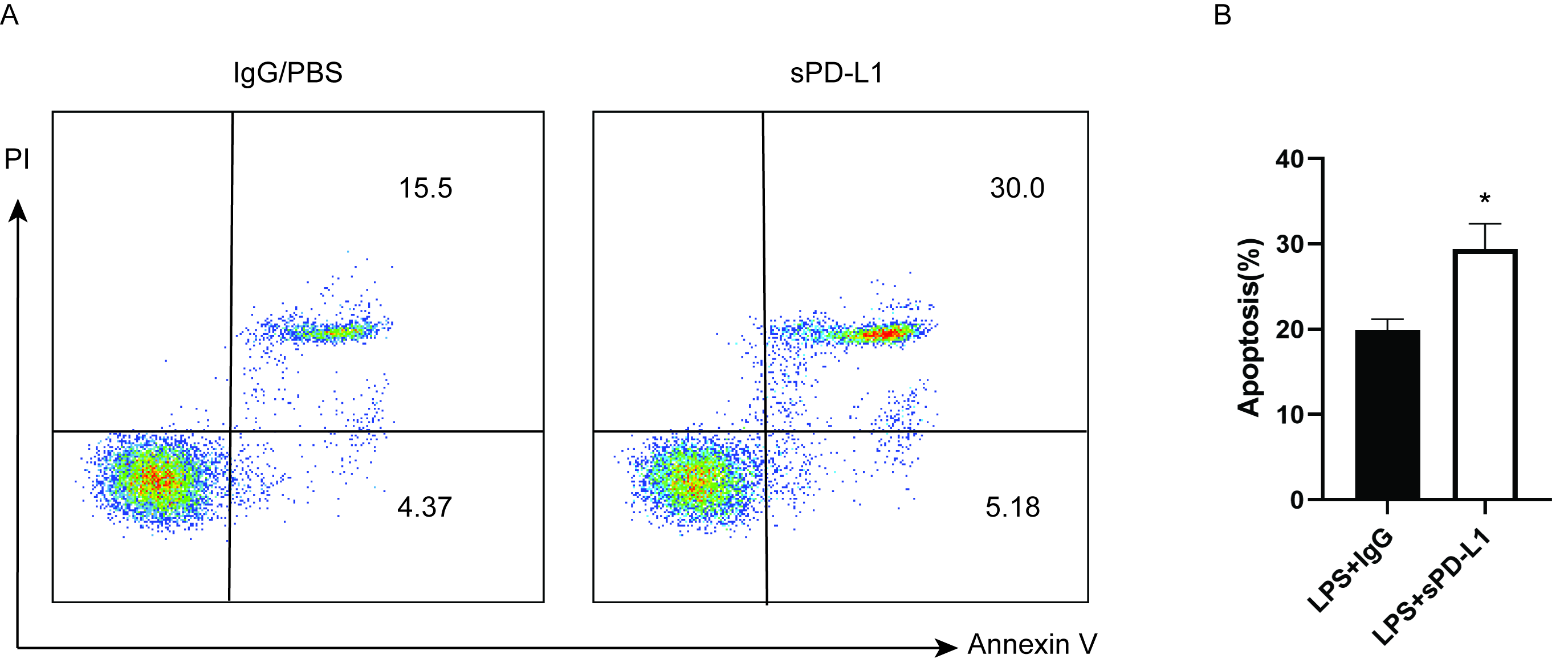

Supplement: Supplementary file 7 — FigureS7 [file 41419_2020_3139_MOESM7_ESM.tif]
