# Supplementary material for: Soluble PD-L1 improved direct ARDS by reducing monocyte-derived macrophages
Source: Cell Death Dis. 2020 Oct 30;11(10):934. doi: 10.1038/s41419-020-03139-9 (PMC7596316; doi:10.1038/s41419-020-03139-9)
Supplement: Supplementary file 8 — Supplementary table1 [file 41419_2020_3139_MOESM8_ESM.docx]

|  | ARDS | | |  |
| --- | --- | --- | --- | --- |
|  | Total | Survivors | Non-survivors | P value |
| N | 44 | 32 | 12 |  |
| Age (year) | 60.83±13.35 | 58.9±12.4 | 63.62±14.13 | NS |
| Gender, male，n(%) | 34（77.3%） | 15 (75.0) | 8 (80,0) | NS |
| Cause of ARDS，n(%) |  |  |  |  |
| *Direct ARDS* | 30 (68.1%) | 20(62.5) | 10(83.3) | NS |
| Pneumonia | 30 (68.1%) | 20(62.5) | 10(83.3) | NS |
| *Indirect ARDS* | 14 (31.8%) | 12(37.5) | 2(16.7) | NS |
| Sepsis or septic shock | 5 (11.3%) | 4(12.5) | 1(8.3) | NS |
| Surgical strike | 6 (13.6%) | 5(15.6) | 1(8.3) | NS |
| Non-septic shock | 1 (2.3%) | 1(3.1) | 0 | NS |
| Pancreatitis | 1 (2.3%) | 1(3.1) | 0 | NS |
| Neuromuscular disease | 1 (2.3%) | 1(3.1) | 0 | NS |
| APACHE II | 16.46±6.7 | 12.9±6.8 | 21.3±5.5 | <0.01 |
| PaO_2_/FiO_2_ | 152.2±66.9 | 143.2±48.0 | 91.9±36.8 | 0.04 |
| CRP, mg/dL | 91.2±93.1 | 66.7±67.5 | 120.2±128.8 | 0.04 |
| PCT, ng/mL | 9.7±18.1 | 4.5±9.8 | 3.2±5.4 | NS |
| Leukocytes, ×10^9^/L | 12.8±7.4 | 10.6±4.7 | 15.5±6.9 | NS |
| Neutrophils, ×10^9^/L | 5.6±5.8 | 3.7±3.3 | 5.8±6.4 | NS |
| Lymphocytes,×10^9^/L | 0.5±0.6 | 0.4±0.4 | 0.4±0.4 | NS |
| VFD, days | 15.6±9.9 | 18.1±8.9 | 9.1±9.9 | NS |
| ICU stay, days | 37.6±44.0 | 34.6±41.3 | 57.8±66.5 | 0.05 |
