# Supplementary material for: Soluble PD-L1 improved direct ARDS by reducing monocyte-derived macrophages
Source: Cell Death Dis. 2020 Oct 30;11(10):934. doi: 10.1038/s41419-020-03139-9 (PMC7596316; doi:10.1038/s41419-020-03139-9)
Supplement: Supplementary file 9 — Supplementary table2 [file 41419_2020_3139_MOESM9_ESM.docx]

| **List** | **Label** | **Marker** | **clone** | **Brand** | **Cat#** |
| --- | --- | --- | --- | --- | --- |
| 1 | 89Y | CD45 | 30-F11 | Biolegend | 103102 |
| 2 | 115In | CD3e | 145-2C11 | Biolegend | 100302 |
| 3 | 139La | h/m-Ki67 | SolA15 | eBioscience™ | 14-5698-82 |
| 4 | 141Pr | CD192(CCR2) | 475301 | R&D | MAB55381-100 |
| 5 | 142Nd | MHC II | Y3P | Bio-Xcell | BE0178 |
| 6 | 143Nd | CD335(NKp46) | 29A1.4 | Biolegend | 137625 |
| 7 | 144Nd | h/m-T-bet | 4B10 | Biolegend | 644802 |
| 8 | 145Nd | CD163 | S15049I | Biolegend | 155302 |
| 9 | 147Sm | Ly6G | IA8 | Biolegend | 127602 |
| 10 | 148Nd | Ter119 | Ter-119 | Biolegend | 116202 |
| 11 | 149Sm | CD64 (FcγRI) | X54-5/7.1 | Biolegend | 139302 |
| 12 | 150Nd | CD14 | Sa14-2 | Biolegend | 123302 |
| 13 | 151Eu | CD161c(NK1.1) | PK136 | Biolegend | 108702 |
| 14 | 152Sm | CD11c | N418 | Biolegend | 117302 |
| 15 | 153Eu | TCRgd | GL3 | Biolegend | 118101 |
| 16 | 154Sm | CD62L | MEL-14 | Biolegend | 104402 |
| 17 | 155Gd | CD103 | 2E7 | Biolegend | 121402 |
| 18 | 156Gd | MCP-1 | 2H5 | Biolegend | 505902 |
| 19 | 157Gd | CD25 | 3C7 | Biolegend | 101902 |
| 20 | 158Gd | CD19 | 6D5 | Biolegend | 115502 |
| 21 | 159Tb | F4/80 | C1:A3-1 | BioRAD | MCA497G |
| 22 | 160Gd | CD274(PD-L1) | 10F.9G2 | Biolegend | 124302 |
| 23 | 161dy | iNOS | CXNFT | eBioscience™ | 14-5920-82 |
| 24 | 162Dy | CD206 | C068C2 | Biolegend | 141702 |
| 25 | 163Dy | IL-6 | MP5-20F3 | Biolegend | 504502 |
| 26 | 164Dy | CD86 | GL-1 | Biolegend | 105002 |
| 27 | 165Ho | IFN-r | XMG1.2 | Bio-Xcell | BE0055 |
| 28 | 166Er | h/m-Arg-1 | E-2 | Santa Cruz Biotechnology | sc-271430 |
| 29 | 167Er | IL-1β | B122 | BD | 550605 |
| 30 | 168Er | Foxp3 | FJK-16s | eBioscience™ | 14-5773-82 |
| 31 | 169Tm | CD273(PD-L2) | TY25 | Biolegend | 107202 |
| 32 | 170Er | CD169 (siglec-1) | 3D6.112 | Biolegend | 142402 |
| 33 | 171Yb | h/m-Gata-3 | TWAJ | eBioscience™ | 14-9966-82 |
| 34 | 172Yb | RORg | 600214 | R&D | MAB6109 |
| 35 | 173Yb | IL-10 | JES5-16E3 | Biolegend | 505002 |
| 36 | 174Yb | CD152(CTLA-4) | UC10-4B9 | Biolegend | 106302 |
| 37 | 175Lu | Siglec-F | E50-2440 | BD | 552125 |
| 38 | 176Yb | TNF-a | MP6-XT22 | Biolegend | 506302 |
| 39 | 197Au | CD4 | RM4-5 | Biolegend | 100520 |
| 40 | 198Pt | CD8a | 53-6.7 | Biolegend | 100716 |
| 41 | 209Bi | h/m-CD11b | M1/70 | PLT |  |
