# Supplementary material for: Soluble PD-L1 improved direct ARDS by reducing monocyte-derived macrophages
Source: Cell Death Dis. 2020 Oct 30;11(10):934. doi: 10.1038/s41419-020-03139-9 (PMC7596316; doi:10.1038/s41419-020-03139-9)
Supplement: Supplementary file 10 — Supplementary figure legend [file 41419_2020_3139_MOESM10_ESM.docx]

**TableS1. Demographics of ARDS patients and controls.**

A:Quantitative data are presented as mean ± SD, Qualitative data are presented as number (%),P-value for the survivors and non-survivors of ARDS; APACHE, acute physiologic and chronic health evaluation; CRP, C-reactive protein; PCT, procalcitonin, MV mechanical ventilation. VFD Ventilator free days.

**Table S2. The cocktail of 41 markers used in mass cytometry**

**Figure S1. Serum sPD-1 and other cytokines in survivors, non-survivors of direct ARDS and healthy controls.**(A) Serum soluble programmed cell death protein 1 (sPD-1) in survivors (n=32), non-survivors (n=12) of total ARDS (n=44) and healthy controls(n=10). (B) Serum soluble programmed cell death ligand-1 (sPD-L1) in survivors (n=32), non-survivors (n=12) of total ARDS (n=44) and healthy controls (n=10). (C) Serum sPD-1 in survivors (n=20), non-survivors (n=10) of direct ARDS (n=30) and healthy controls (n=10). (D) Serum IL-10 in survivors (n=20), non-survivors of direct ARDS and healthy controls. (E) Serum IL-17 in survivors (n=20), non-survivors (n=10) of direct ARDS (n=30) and healthy controls (n=10). All the data were compared by Mann-Whitney U-tests.

**FigureS2. Receiver-operating characteristic curve (ROC) for serum sPD-L1 level and APACHEII for predicting ICU mortality of patients with direct ARDS (n = 30).**

**Figure S3. X-shift analysis of CD45+ live cells.** A)The heatmap of the expression of 41 markers in all clusters. B)The frequency of 49 clusters in CD45+ cells analyzed by X-shift.

**Figure S4. The kinetics of the distribution sPD-L1 in lung.** A) The distribution of sPD-L1-His was evaluated at the protein level by immunoblotting (n=4/group). B）The relative protein expression levels of PD-L1-His C) The immunohistochemical staining of sPD-L1 fused His protein in lung of ARDS mice without sPD-L1-His injection (control) or at 1h post injection of sPD-L1-His protein (1h). *P < 0.05, **P < 0.01, ***P < 0.001, analyzed by t test.

**Figure S5. The bacterial load and levels of TNF-α in BALF of ARDS mice treated with sPD-L1 or IgG.** A) The bacterial load in BALF of ARDS mice treated with sPD-L1 or IgG (n=9/group) B) The levels of TNF-α in BALF of ARDS mice treated with sPD-L1 or IgG (n=9/group) B). Each value represents the mean ± SEM of one of the three independent experiments. *P < 0.05, **P < 0.01, ***P < 0.001, analyzed by t test.

**Figure S6 The proliferation and chemotaxis of macrophages did not change after administration of sPD-L1.** A) The expression of ki67 in lung cells. Red circle represents the monocyte-derived macrophages. B) The percent of ki67 postive cells in monocyte-derived macrophages. C) The levels of MCP-1 in serum.n=4/group

**Figure S7. The apoptosis of peritoneal macrophages** **treated with sPD-L1 or IgG. A)** The apoptosis of macrophages by PI/Annexin-V staining. D) Apoptosis of peritoneal macrophages treated with sPD-L1 or IgG.(n=3/group), analyzed by annexin V-fluorescein isothiocyanate/PI double staining. Cells in the B2 and B4 quadrants (annexin V+/PI+ and annexin V+/PI-, respectively) were considered to be apoptotic. D) Graphical representation of apoptosis (n=3/group). Each value represents the mean ± SEM of one of the three independent experiments. *P < 0.05, **P < 0.01, ***P < 0.001, analyzed by t test.
